# Challenges of transsphenoidal pituitary surgery in severe brachycephalic dogs

**DOI:** 10.3389/fvets.2023.1154617

**Published:** 2023-06-20

**Authors:** Lucinda L. Van Stee, Sarah J. Van Rijn, Sara Galac, Björn P. Meij

**Affiliations:** ^1^Small Animal Surgery, Department of Clinical Sciences, Faculty of Veterinary Medicine, Utrecht University, Utrecht, Netherlands; ^2^Small Animal Internal Medicine, Department of Clinical Sciences, Faculty of Veterinary Medicine, Utrecht University, Utrecht, Netherlands

**Keywords:** cushing, pituitary, surgical oncology, endocrinology, neurosurgery, adenoma, brachycephalic, brachycephalic airway obstructive syndrome (BAOS)

## Abstract

**Introduction:**

Transsphenoidal hypophysectomy is the standard surgical technique for the excision of pituitary neoplasms. Anatomy may be more obscured in brachycephalic skull types due to the crowding of soft tissue and osseous structures. We describe the unique challenges to approach the sphenoid bone and localize the correct burr hole site in severe brachycephalic dogs.

**Materials and methods:**

A single institution retrospective case series of brachycephalic dogs with pituitary-dependent hypercortisolism (PDH). Preoperative computed tomography enabled 3D-, and cross-sectional reconstruction to plan and dry-practice the position of the ideal burr hole in relation to the sella turcica, pterygoid hamular processes, and hard palate. Rostral burring of the caudal hard palate obscuring the direct sphenoid approach necessitated adaptations to the original transsphenoidal hypophysectomy procedure. Postoperative outcomes and complications with respect to those seen in mesocephalic dogs are described.

**Results:**

Ten brachycephalic dogs including French Bulldogs (*n* = 9) and a single Dogue de Bordeaux were included. All dogs were diagnosed with PDH and had preoperative advanced imaging performed on the skull. All but one dog had an enlarged pituitary gland, with a median pituitary/brain value of 0.5 (range 0.21–0.9). A total of 11 transsphenoidal hypophysectomy procedures were performed in these 10 dogs. Rostral extension of the soft palate incision into the hard palate was performed to access the burr hole site on the sphenoid bone. Major complications included aspiration pneumonia (*n* = 1), severe gastroesophageal reflux (*n* = 1), and central nervous signs (=1). All dogs survived until discharge, with a median time to follow-up of 618 days (range 79–1,669 days). Seven dogs experienced long-term remission of PDH.

**Conclusion:**

Brachycephalic dogs undergoing transsphenoid al hypophysectomy benefit from meticulous presurgical planning and extension of the approach into the caudal hard palate. Advanced surgical skills can render a good outcome in a technically challenging environment.

## 1. Introduction

Transsphenoidal hypophysectomy is a procedure used for the surgical treatment of pituitary neoplasms in dogs and cats. This technique is described by Meij et al. (1) and has been performed on over 430 canine and 30 feline patients referred to the Department of Clinical Sciences of the Faculty of Veterinary Medicine at Utrecht University since the publication of the technique, with good results (2–5) and defined prognostic parameters (3, 4, 6–8).

At this moment, the technique is only performed by a handful of veterinary surgeons around the world (UK, Italy, Japan, USA) (9–13). This is due to the surgical challenges of identifying the exact location of the pituitary gland to determine the burr hole site and trajectory, as well as the requirements of an endocrinology division open to surgical treatment and the need for specialized anesthesiology and postoperative intensive care. Another factor complicating the exact localization of the burr hole in the transsphenoidal approach is the great variety in dog skull shapes and sizes among the cohort of patients referred for pituitary surgery. Especially the brachycephalic and dolichocephalic skull morphology poses additional challenges to the surgical approach.

The aim of this study is to describe the surgical approach and outcome of brachycephalic patients undergoing transsphenoidal hypophysectomy.

## 2. Material and methods

### 2.1. Study design

In this single-institute retrospective study, we describe a subset of patients with pituitary-dependent hypercortisolism (PDH) and brachycephaly, referred to us from 1993 to 2022.

#### 2.1.1. Patient information

The medical records of all canine cases that underwent a transsphenoidal hypophysectomy procedure at our hospital were evaluated retrospectively, and brachycephalic dogs were included in this study. Patient signalment (breed, sex, age, and body weight), history, preoperative medical diagnosis, physical examination findings at the time of admission prior to surgery, diagnostic imaging, laboratory tests, surgical procedures performed, perioperative and postoperative complications, tumor histology and immunohistology, and patient outcome were analyzed.

We included 10 dogs with PDH and severe brachycephaly in this study. The diagnosis was made on the dog's medical history and clinical signs, and confirmed by endocrine testing, either low-dose dexamethasone suppression test (LDDST) or urinary corticoid creatinine ratio (UCCR) combined with the high-dose dexamethasone suppression test (HDDST). Preoperative endogenous ACTH concentration was measured in all dogs after 2007 and supported the diagnosis of PDH. All cases referred for surgical treatment over this timeframe were included, irrespective of their conformation or BOAS. No dogs were denied the transsphenoidal hypophysectomy procedure based on their BOAS in this study. Other brachycephalic dogs such as Boxer dogs and English Bulldogs were also seen in this time frame; however, their skull conformation did not necessitate the adaptations to the transsphenoidal approach, that are described in this study and were therefore not included.

#### 2.1.2. Preoperative diagnostic imaging of the pituitary gland and adrenal glands

In all dogs, a CT scan was performed to visualize the pituitary and adrenal glands. Advanced imaging was performed to evaluate the size of the pituitary gland and to screen for co-existence with an adrenocortical tumor as described in previous publications (13, 14) and to choose the most optimal treatment option. The pituitary height/brain area (P/B) value was used to relate the size of the pituitary gland to the size of the dog's skull (15). A P/B value exceeding 0.31 was indicative of an enlarged pituitary gland.

#### 2.1.3. Postoperative diagnostic imaging of the pituitary fossa

The indication, imaging diagnosis, and outcome of two cases involving postoperative imaging of the pituitary fossa were reported.

#### 2.1.4. Preoperative surgical planning

**Figure 1:**
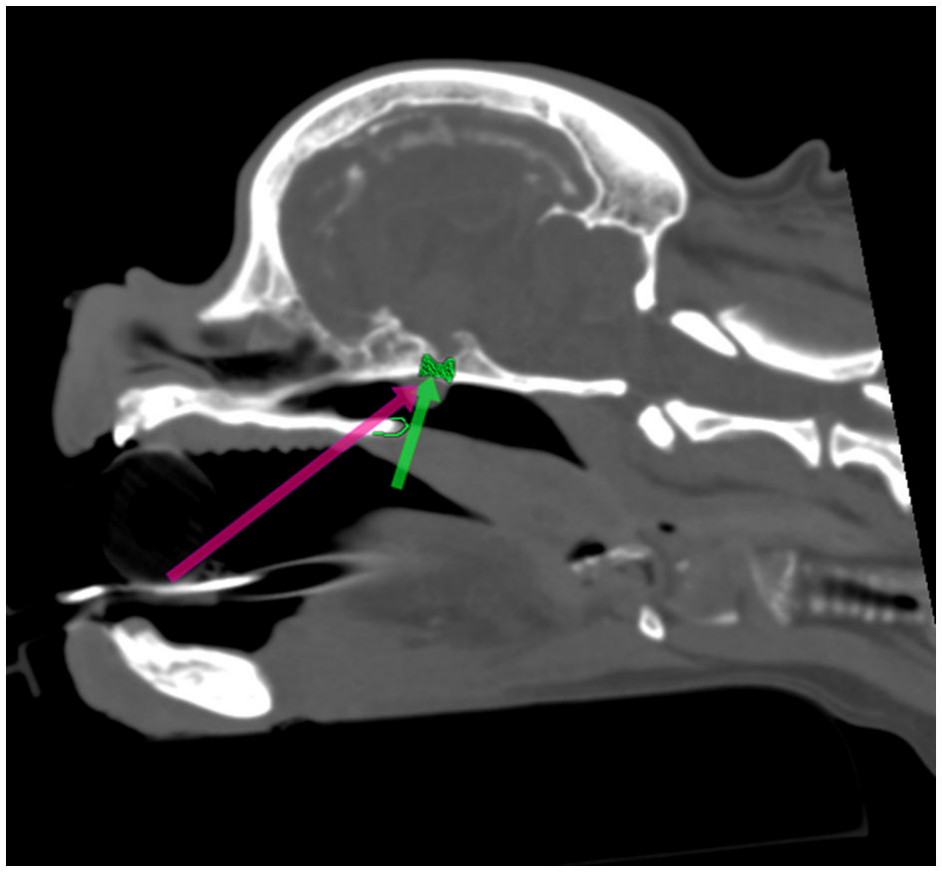
Presurgical planning through multiplanar and 3D reconstruction with *in silico* practice based on a preoperative CT scan (Figures 1–5). The 90 degrees angle to the sphenoid bone at the level where the sphenoid flattens is marked by the green arrow. The planned burr hole is marked green on the bone. The pink arrow shows the surgeons' view of the sphenoid bone.

**Figure 2:**
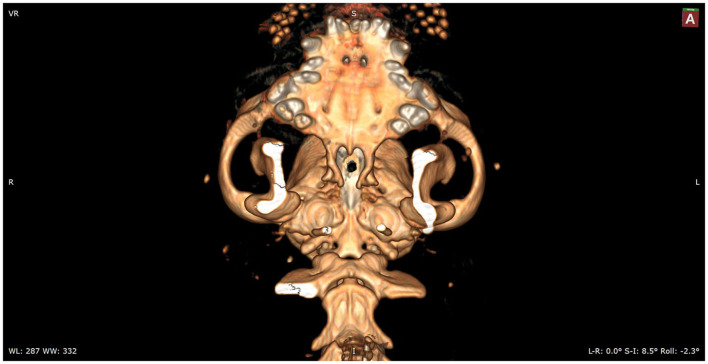
*In silico* practice of the burr hole location. A 3D reconstruction is made in which the planned burr hole is made. This view shows the burr hole trajectory in a straight view.

**Figure 3:**
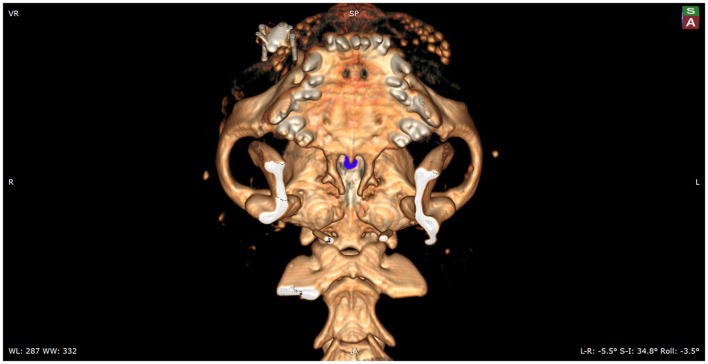
Mockup of the actual intraoperative view of the sphenoid bones. The view onto the burr hole, which is marked with blue, is partially obscured by the hard palate. To enable the surgeon a full visualization of the sphenoid bones, the caudal aspect of the hard palate will need to be removed.

**Figure 4:**
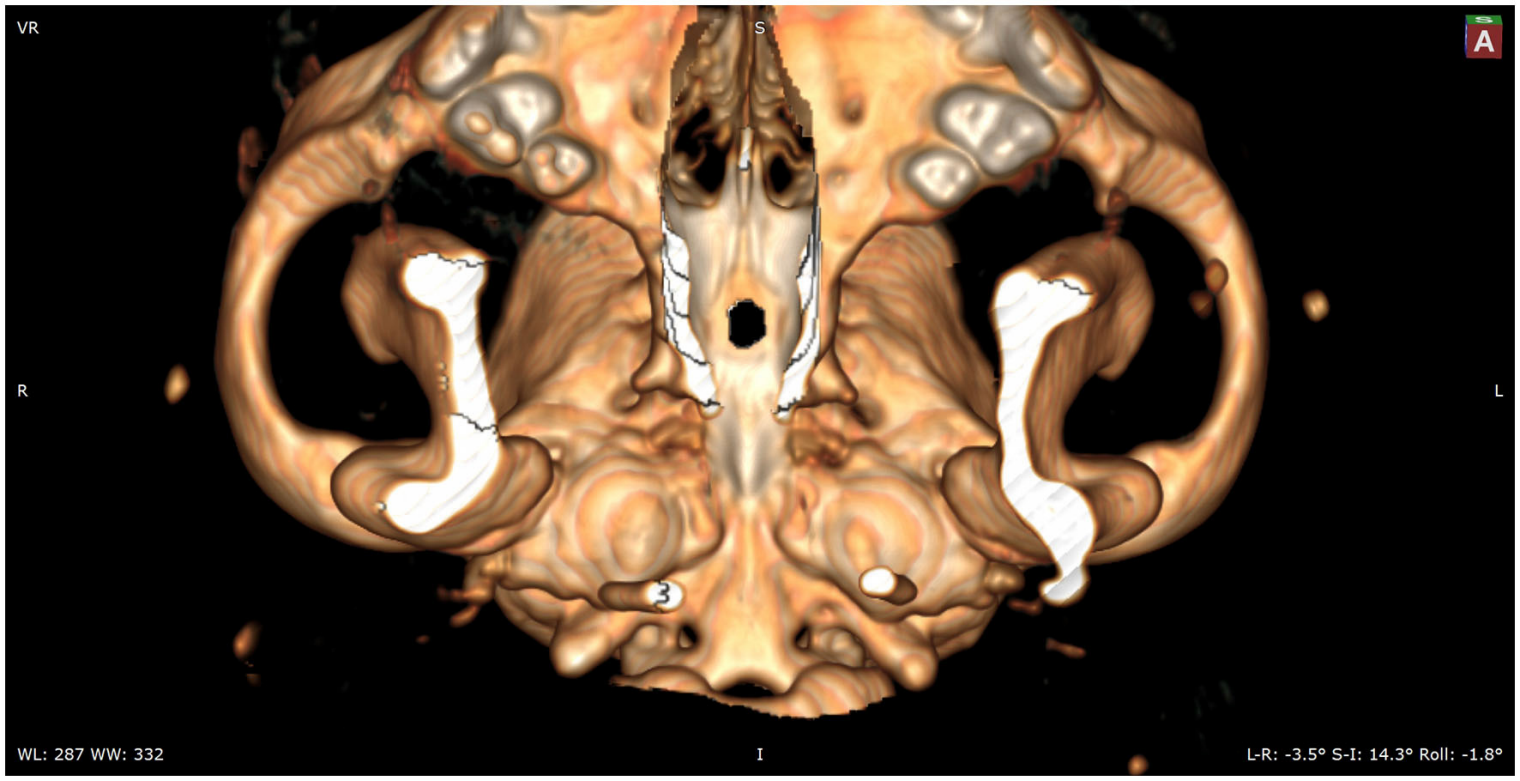
In this image, the caudal aspect of the hard palate is removed to show the full sphenoid and part of the vomer bones.

**Figure 5:**
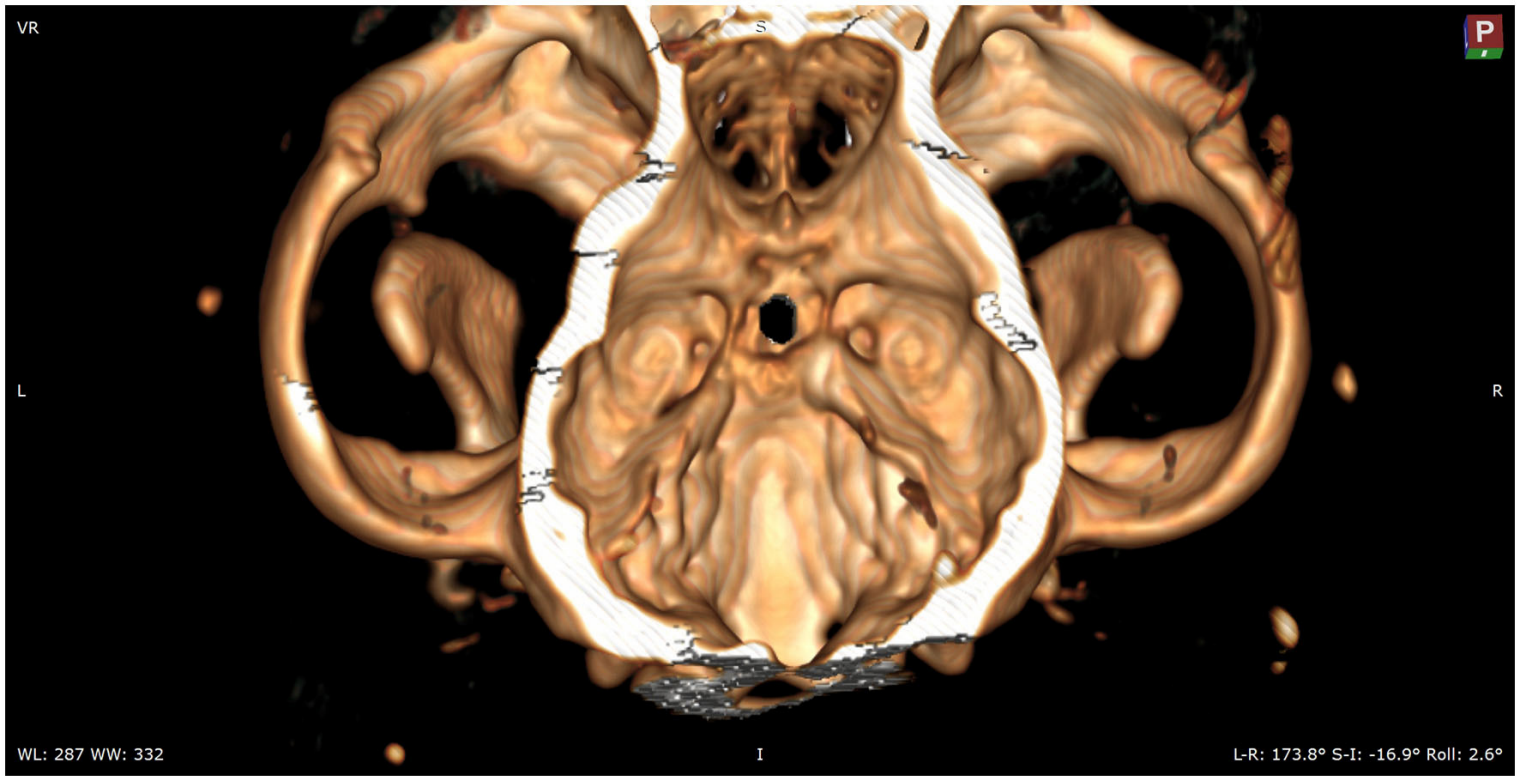
Assessment of the placement of the burr hole by evaluating the hole within the sella.

Following preoperative diagnostic imaging, surgical planning was initiated. Patient-specific anatomical landmarks and burr hole placement were determined based on sagittal and transverse multiplanar reconstructions of the advanced diagnostic imaging performed. Planning included the location of the center of the pituitary gland and the distance between surgical landmarks, including the caudal extension of the hamular processes, the tuberculum sellae, the pituitary fossa, the rostral part of the dorsum sellae, and the area where the sphenoid bone changes its nasopharyngeal surface from a ridge, to flat, to a groove, as previously described (1) (Figure 1). Starting from 2013, in a total of six cases, a 3D reconstruction was made using the Medixant RadiAnt DICOM Viewer. The sphenoid bone was approached *in silico*, imitating real-life surgery (Figures 2–4). For this dry practice, the mandible and tracheal tube and tongue bones were erased giving an unobstructed view of the sphenoid bone. Based on the set surgical landmarks, a safe burr hole was planned and executed *in silico* using the surgical blade tool in the RadiAnt DICOM Viewer (Medixant) (Figures 3, 4). The location of the burr hole was inspected inside the neurocranium by erasing the dorsal part of the calvarium exposing the complete skull base from the inside of the neurocranium (Figure 5). In the correct approach, the burr hole was situated exactly in the midline between the tuberculum sellae and the dorsum sellae at the level of the pituitary fossa (Figure 5).

### 2.2. Surgical technique and perioperative care

The transsphenoidal hypophysectomy procedure, as performed in all cases, was based on the microsurgical technique previously described (1). The approach was modified based on the brachycephalic skull anatomy, and differences in perioperative complications were identified and described in detail in the result section of this study. Data regarding perioperative care and complications during both surgery and hospitalization were extracted from the medical records and evaluated for anomalies/differences compared to previous reports.

After surgery, all dogs were treated through a hormone substitution protocol that has been described previously (4). After discharge, the remission of PDH was monitored by the measurements of UCCRs at 2 weeks, 8 weeks 6 months, 12 months, and every year for the rest of their lives. The interval between testing may be shortened if there was doubt about possible recurrent PDH.

### 2.3. Postoperative outcome

Histological and immunohistochemical diagnoses, complications, and overall outcome and survival were reported.

Outcome information was collected from the longitudinal follow-up and where needed, telephonic and email consultations with owners and referring veterinarians were conducted at the time of writing this article.

Residual disease was defined when there was obvious incomplete resection at the time of surgery, plasma ACTH concentration levels within hours after surgery were continuously elevated, and/or the UCCR exceeded the cutoff level of 10 × 10^−6^ and clinical signs of hypercortisolism were present within 8 weeks after surgery (4). Remission was considered if UCCR <10 × 10^−6^, and the resolution of clinical signs of hypercortisolism was achieved.

Postoperative mortality was defined as death within 4 weeks after surgery irrespective of cause of death (4). Survival times and time to follow-up were calculated from the surgery date up to either the last contact moment with the owner or the registered date of death. Statistical analysis was performed using SPSS 27.0 (IBM, Armonk, New York).

## 3. Results

### 3.1. Patient characteristics

Ten brachycephalic dogs were included in this study. There were two breeds present in this group: French Bulldogs (*n* = 9) and a single Dogue de Bordeaux. Most dogs were neutered, with four spayed female dogs, five castrated males, and a single intact male dog. The median age was 6.9 years (range 4.4–8.7 years), the median body weight was 11.7 kg (range 9.9–59.8 kg).

Primary clinical signs were associated with hypercortisolism, including polyuria/polydipsia (*n* = 10), severe polyphagia (*n* = 10), central obesity (*n* = 10), lethargy (*n* = 10), a dull fur coat (*n* = 10), flank alopecia (*n* = 10), and calcinosis cutis (*n* = 2). A single case demonstrated a decreased mental status, and another case was previously diagnosed with primary epilepsy. In all dogs, the diagnosis of PDH was confirmed with endocrine testing (Table 1). Out of 10 dogs received trilostane in the preoperative period. A single dog (case 6) was initially started on the medical management of PDH caused by a non-enlarged pituitary but failed medical management and was therefore referred. The initial P/B value was 0.39 and increased to 0.9 at the time of referral to our hospital, 8 months after initiating trilostane therapy.

**Table 1:** Patient-specific details.

**Patient number**	**Breed**	**Age**	**Sex**	**Preoperative functional test performed**	**Preoperative endogenous ACTH**	**Postoperative endogenous ACTH**	**Postoperative UCCR at 8 weeks**	**Last UCCR [time]**	**Status at the time of follow-up**	**Follow-up**	**Time to follow-up [days]**
1	French bulldog	7	FC	UCCR + HDDST			5.1	5.1 [8 weeks]	D	R	79
2	French bulldog	7	MC	UCCR + HDDST	154	16.3	0.5	0.5 [8 weeks]	D	R	1,080
3	French bulldog	7	MI	Baseline UCCR, LDDST, endogenous ACTH	146	29.8	9.4	9.4 [8 weeks]	D	RD	177
4	French bulldog	6	FC	UCCR + HDDST	48		0.4	1.2 [2 years]	D	R	1,237
5	French bulldog	7	FC	UCCR + HDDST	179	10.7	0.6	1.5 [4 years]	A	R	1,669
6	French bulldog	6	MC	Endogenous ACTH, LDDST	756	8.5	1.6	1.6 [6 months]; 107.75 [1 year]	A	RD	771
7	French bulldog	8	MC	Single UCCR, LDDST	36	10.5			A	R	695
8	French bulldog Sx 1	4	MC	LDDST	787	201		65 [6 months]	A	RD	580
8	French bulldog Sx 2	5	MC	UCCR baseline	147	52			A	R	241
9	French bulldog	9	MC	UCCR + HDDST	15	6.3			A	R	201
10	Dogue de Bordeau	5	FC	UCCR + HDDST	67	10	0.2	0.2 [1 year]	D	R	618

FI, female intact; FC, female castrated; MI, male intact; MC, male castrated. Functional testing includes: baseline serum ACTH; UCCR, urine corticoid creatinine ratio; HDDST, high-dose dexamethasone suppression test; LDDST, low-dose dexamethasone suppression test; R, remission; RD, recurrent disease; D, deceased; A, alive.

### 3.2. Imaging results

All cases had a CT scan performed in the preoperative setting. Three of these CT scans were performed abroad but were available for review prior to surgery. One case also had an MRI performed in the diagnostic work up, which was performed abroad. Only one case that had its imaging performed at an external clinic needed repeat imaging for surgical planning. In the case of the Dogue de Bordeaux, the initial CT scan did not include enough of the hard palate to plan the surgical procedure and was therefore repeated prior to surgery. Three cases had postoperative diagnostic imaging of the pituitary fossa performed. The case which had revision surgery after 11 months for tumor regrowth had a repeat CT scan performed 1 day prior to the second pituitary surgery procedure. One case had an MRI performed due to recurrent polyuria/polydipsia at 28 months after surgery, without other signs or functional testing results pointing to recurrent PDH. The dose of hydrocortisone acetate was lowered, and clinical signs of polydipsia/polyuria subsided. The third case had a postoperative MRI scan performed 17 days after the procedure. This patient suffered from severe dehydration caused by ongoing sodium losses. Imaging showed no signs of complications in the thalamic area, pituitary fossa, and hypothalamic area. Desmopressin was not affecting the sodium losses, and aldosterone levels were considered sufficient. It was suspected that there was damage to the hypothalamus, causing an inadequate drinking response.

Nine dogs had an enlarged pituitary gland with a median P/B value of 0.52 (range 0.33–0.9). One dog had a non-enlarged pituitary gland with a P/B value of 0.21. Case-specific tumor dimensions are reported in Table 2. The sphenoid bone had a median thickness of 6.6 mm (range 3–8.5 mm) as measured on the preoperative CT scan in all 11 procedures, including the case of the revision. The median sphenoid bone thickness in all primary cases (*n* = 10) was 6.7 mm (range 5.7–8.5 mm). In case 8, the sphenoid bone was measured 3 mm prior to the second hypophysectomy procedure.

**Table 2:** Surgery and tumor-specific details.

**Case ^#^**	**Procedure ^#^**	**P/B value**	**Sphenoid; Bone; Height [mm]**	**Length hard palate osteotomy [mm]**	**Intraoperative complications**	**Surgery time (minutes)**	**Postoperative complications**	**Difficulty; Grade (note 1)**	**H&E staining; histology**	**Infiltration**	**ACTH**	**GH**	**aMSH**
1	1	0.21	5.7					1	Pituitary adenoma		+	-	-
2	2	0.4	6.1	20	First approach attempt failed			1	Pituitary adenoma	+	+	-	-
3	3	0.63	7.8	10		192	Tracheostomy Pneumonia; Enucleation OD	1	Pituitary adenoma	+	+	-	+
4	4	0.44	7.6	15		176	Reflux esophagitis; Rhinitis	1	Pituitary adenoma	-	+	+	-
5	5	0.52	6.7	10		138	Mild stridor; Transient hyperthermia	1	Pituitary adenoma of pars intermedia; Craniopharyngeal duct cyst	-	+	-	-
6	6	0.9	8.4	10		171	Transient hyperthermia; Nasal discharge; Persistent DI	2	Pituitary adenoma	-	+	-	-
7	7	0.59	7.5	10		138	Severe hyponatremia	4	Pituitary adenoma	-	+	-	-
8	8	0.75	5.8	15		189		2	Pituitary adenoma	+	+	-	-
8	9	0.48	3	15	Severe intraoperative hemorrhage	161	CNS signs	1	Pituitary adenoma	-	+	-	+
9	10	0.33	6.5	15		158	Reflux esophagitis (feeding tube necessary); Palatal dehiscence; Rhinitis	1	Pituitary adenoma	+	+	-	+
10	11	0.36	8.5	20	First approach attempt failed. Fracture of the hamuloid process	189	Nasal discharge; Transient hyperthermia; Mild stridor	1	Pituitary adenoma	-	+	+	+

IHC, immunohistochemistry; DI, diabetes Insipidus.

Grading difficulty of surgical procedure: 1 = difficult, 2 = hard, 3 = mediocre, 4 = relatively easy, 5 = easy.

### 3.3. Surgical alterations to the original technique

**Figure 6:**
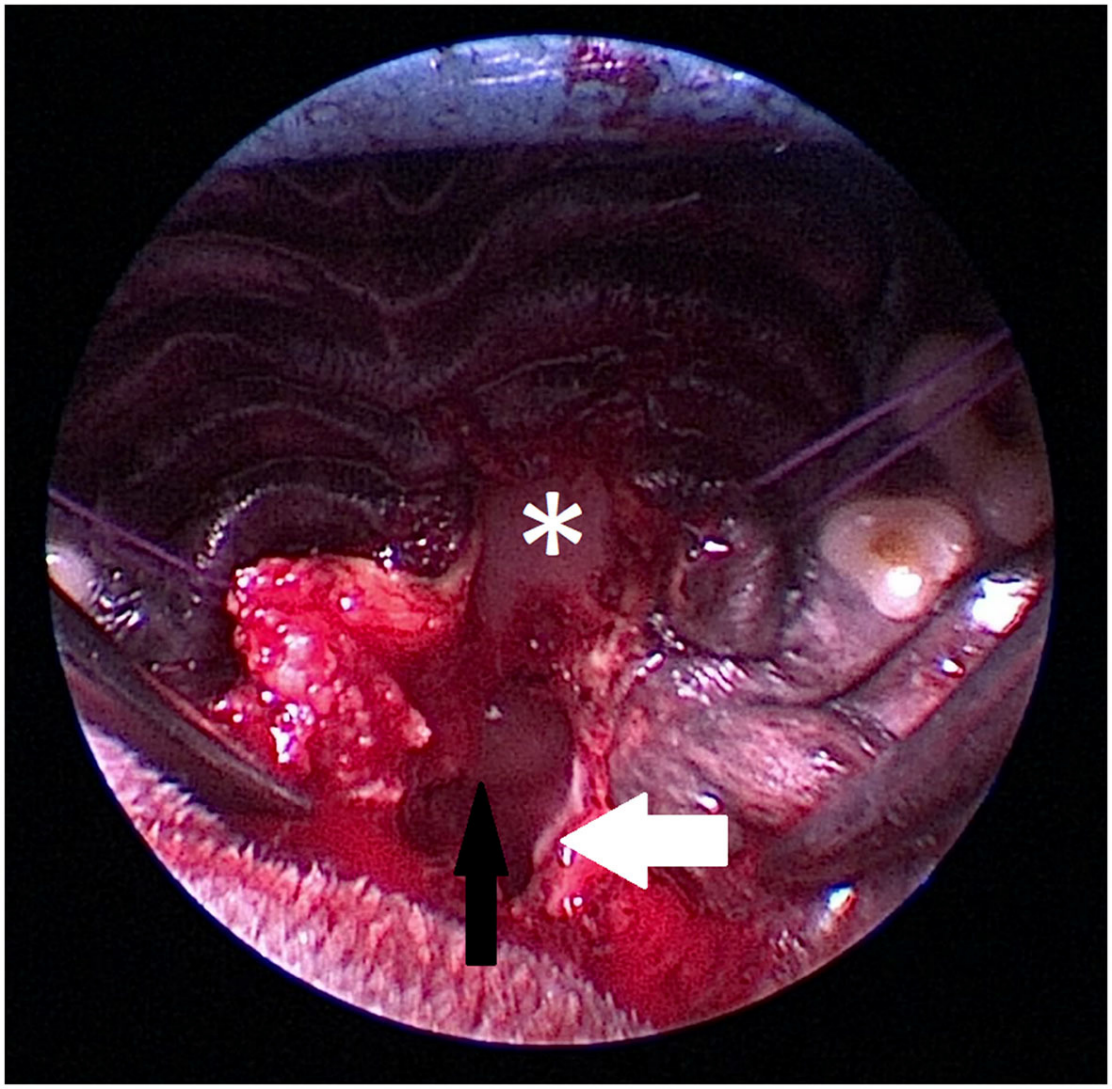
Intraoperative view of anatomic landmarks during the procedure (Figures 6–9). The images show the proximity of the hard palate structures obscuring the view of the sphenoid bone. The hard palate (asterix) is obscuring the view of the presphenoid. The hamular process (white arrow) of the pterygoid bone is marking the caudal anatomic landmark. A craniopharyngeal duct cyst (black arrow) is visible within the nasopharynx.

**Figure 7:**
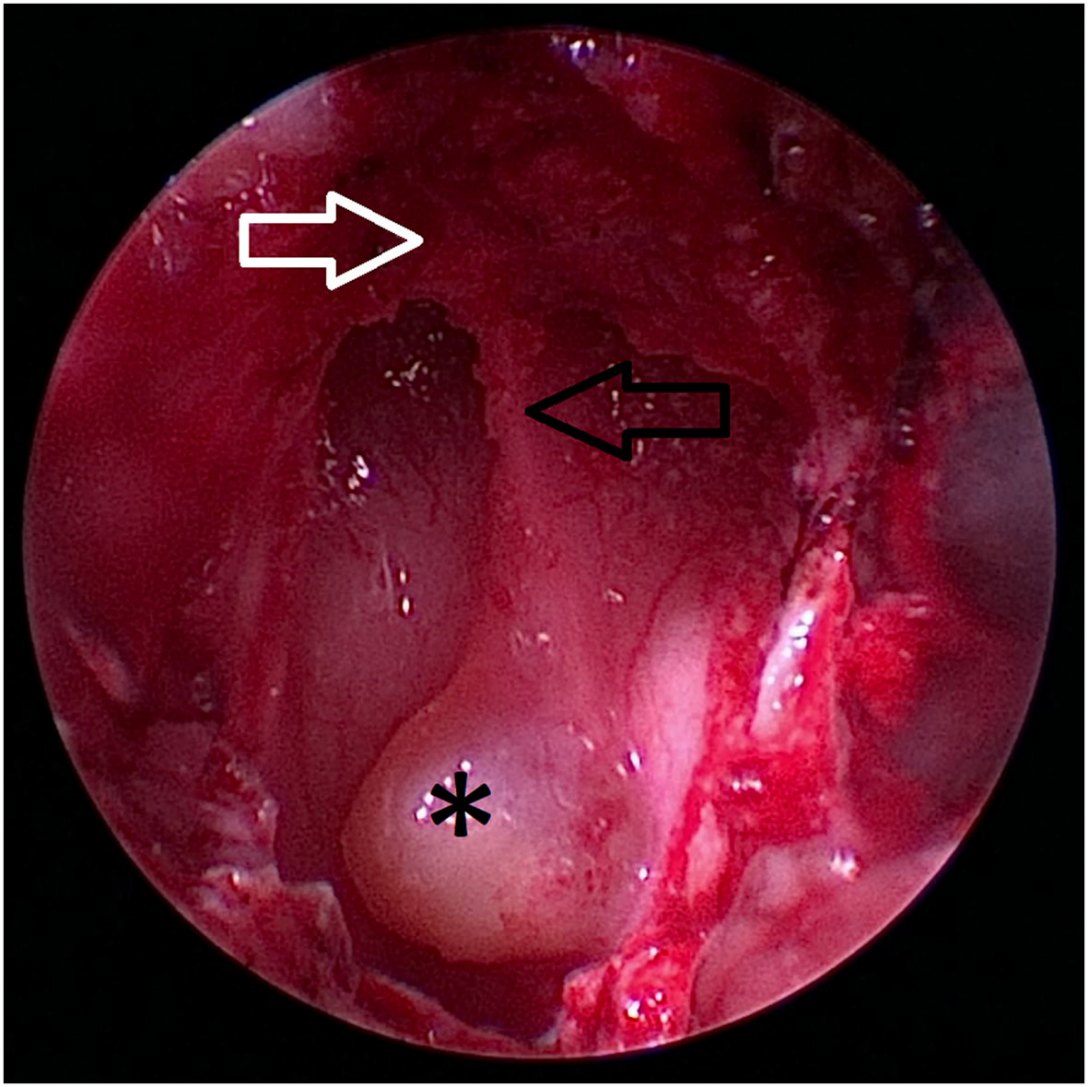
Caudal extent of the hard palate (white arrow) is excised. The nasal septum (black arrow) at the level of the os vomer is still visible (black arrow). The craniopharyngeal duct cyst (asterix) marks the location of the transsphenoidal approach to the sella.

**Figure 8:**
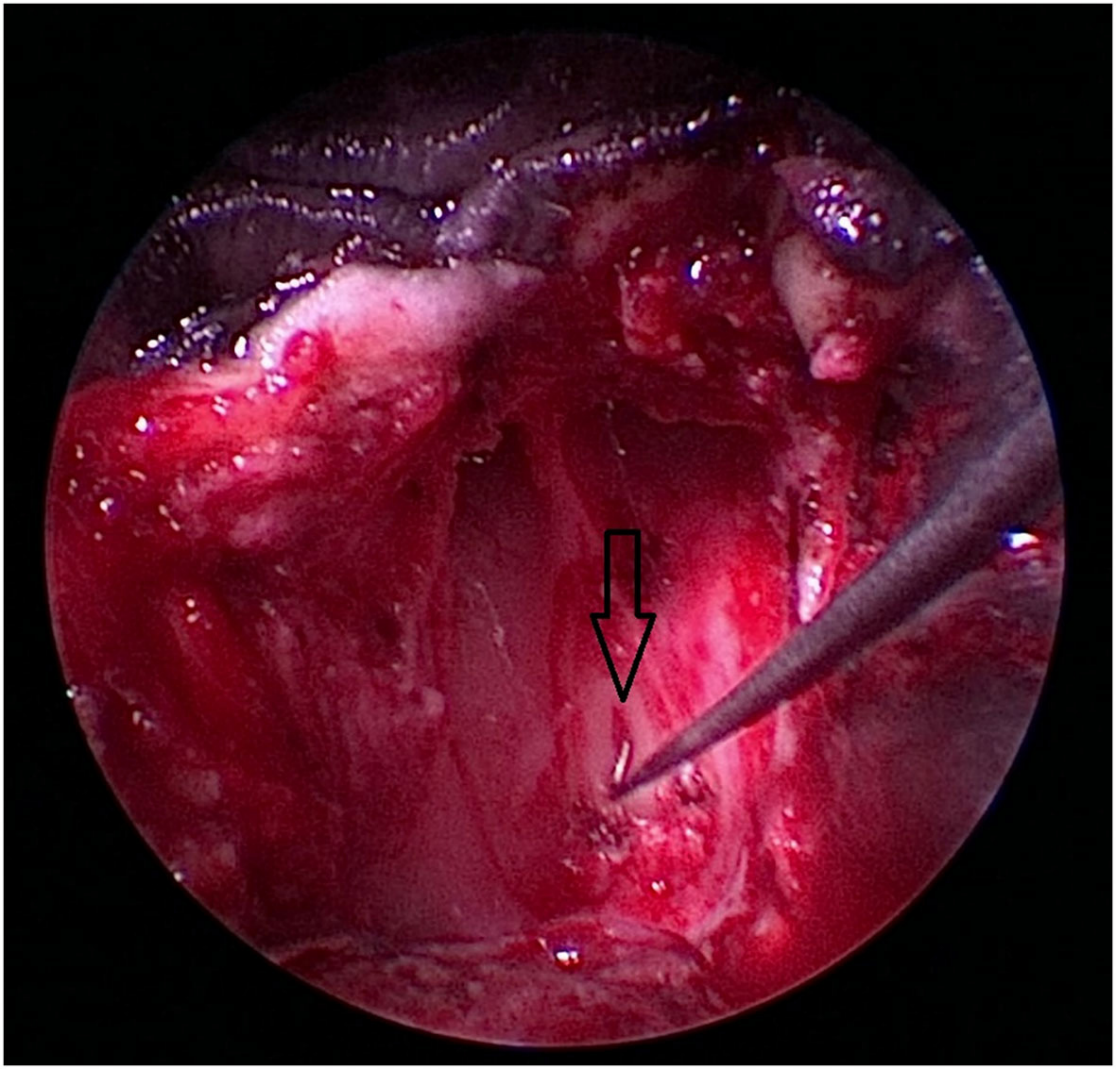
After removal of the craniopharyngeal duct cyst, the opening of the craniopharyngeal duct (black arrow) is visible in this case.

**Figure 9:**
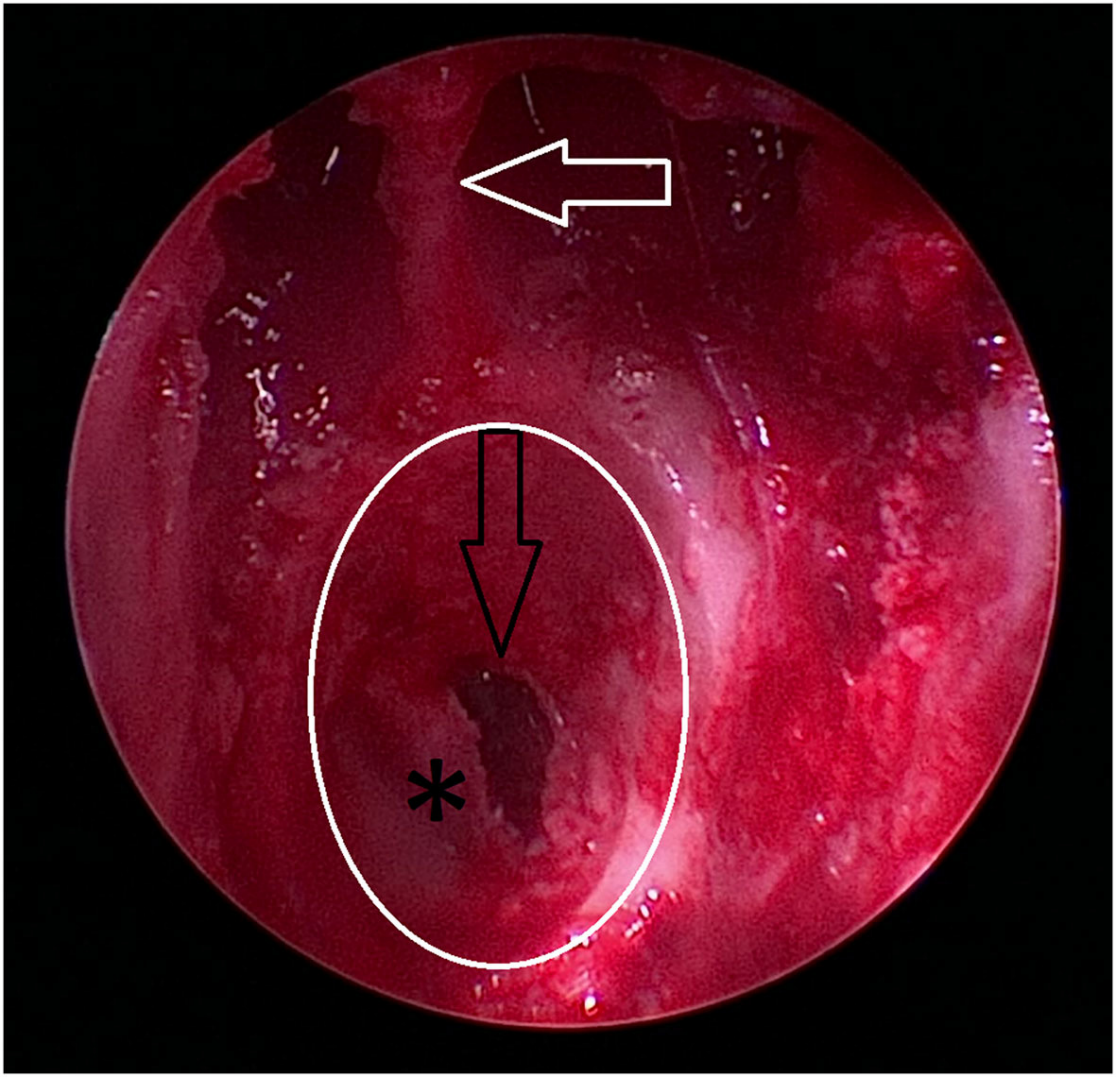
Burr hole (white circle) is completed, and the dura (asterix) is partially incised. The depth of the burr hole is marked by the black arrow. As an anatomic reference, the caudal extent of the nasal septum formed by the os vomer is marked by a white arrow.

In all transsphenoidal hypophysectomies, the standard soft palate approach as previously described (1) had to be extended rostrally in the caudal hard palate [Figure 6], as the planned burr hole site in the sphenoid bone was partially or completely obscured by the hard palate. The midline incision into the soft palate was extended over the hard palate exposing the vomer bone, using a scalpel blade (Figure 6). Stay sutures were placed in the soft palate and the oral mucosa overlaying the hard palate (3-0 polygalactin 910; Vicryl, Ethicon, Norderstedt, Germany) to protect it from injury and increase the visualization of the hard palate. Next, the caudal extension of the hard palate was removed using a high-speed burr, adapting the width and length of the hard palate slot to such a size, that the sphenoid approach would not be hampered by the hard palate (Figures 7–9). The median extension into the hard palate was 12.5 mm (range 10–20 mm) in length. In the initial cases, the length and width of the hard palate slot were determined intraoperatively. In later cases, the length was estimated based on the preoperative advanced imaging and adapted during the procedure if necessary. Based on these 10 cases, a minimum of 1 cm length of the slot should be prepared. The maximum width of the hard palate slot was just slightly wider than the width of the opening within the sphenoid bone, to allow the passage of the burr easily and remain a good view. In one case, lateral fracturing-bending of a hamular process with a Gelpi retractor (case 10) was needed to increase the surgical exposure because of the midline deviation of the pterygoid bones.

After the approach to the pituitary fossa and extraction of the pituitary tumor, the sphenoid burr hole was closed in a routine way (1). The nasopharyngeal hard palate mucoperiosteum was often not recognized anymore and was left to heal by secondary intention. The oral mucosal layer overlying the hard palate mucosa was sutured in the same manner as the soft palate mucosa was closed.

The median duration of a transsphenoidal surgery in this study was 171 min (range 138–192 min) and slightly exceeded the median time of transsphenoidal surgery in dogs in our hospital, which is approximately 120–150 min (unpublished data).

On a 5-point semantic differential scale (1 = difficult, 5 = easy), 8 out of 11 procedures were considered difficult by the experienced surgeon having performed more than 400 hypophysectomies (Table 1). This scale is based on the ability to obtain both a good view and access to the pituitary fossa. Remarks made by the surgeon most often mentioned difficult access due to skull conformation, including crowding of both soft and osseous tissues in the surgical area. The duration of surgery was not indicative of the ease of the surgical procedure.

Intraoperative complications included profuse intraoperative hemorrhage (*n* = 8) and in two cases, the initial surgical procedure was aborted during a first attempt (cases 2 and 10). In both cases in which the first attempt failed, there were discrepancies between the initial presurgical planning and intraoperative anatomical landmarks, leading to difficulties in the identification of the planned burr hole site. In case 2, the initial approach was made too far caudal, and the following dural incision the basilar artery was exposed in the subarachnoid space overlying the pons. In case 10, there was poor visualization of the fossa. The procedure was abandoned in both cases, and the patient closed as per protocol for the planned pituitary surgery. A definitive second surgery was rescheduled and performed within a fortnight and executed without further complications. In both cases, the hard palate was resected over a length of 20 mm, which improved the visibility and access to the sphenoid bone and pituitary fossa sufficiently.

The soft tissues posed another hurdle in our cohort, as the oral cavity was more crowded. In all cases, the soft palate was thicker than seen in non-brachycephalic cases and all cases had macroglossia. An oral inspection to assess the severity of brachycephalic obstructive syndrome was performed. None of the cases received additional surgery of the upper airways during the same anesthesia. All cases either had previous upper airway surgery at a younger age, had no signs of laryngeal collapse, or at least not exceeding grade 1 or recovered from recent previous anesthetic procedures without complications related to their BOAS. In all cases, at the time, the BOAS was not considered too problematic to postpone or cancel the surgery.

In case 8, revision surgery was performed due to the regrowth of the primary tumor. This case was initially presented with central neurological signs and severe PDH. The dog had a severely enlarged pituitary gland, with a P/B value of 0.75. During the first procedure, a massive, partially mucoid mass was removed, without complications at the time of surgery noted. During the approach of the second surgery, almost immediate and profuse hemorrhage was present, which continued throughout the procedure leading to poor intraoperative visibility.

### 3.4. Postoperative period and outcome

All dogs survived the surgical procedures and were discharged from our hospital. The median time of hospitalization was 4.5 days (range 3–11 days). There was no postoperative mortality within 4 weeks after surgery. Of the 10 dogs that were included in this study, five dogs are still alive at the time of follow-up. One of these five cases (case 8) has had two surgeries and is currently only affected by a slight left-sided head turn, without clinical signs of recurrence of the PDH. Long-term follow-up information was available for 9 out of 10 patients. Overall median time to follow up was 618 days (range 79–1,669 days), with a median survival of 618 days for the deceased five cases (range 79–1,237) and a median time to follow up of 695 days (range 201–1,669 days) for the five cases still alive at the time of writing this manuscript. All five deceased dogs were euthanized. Causes of euthanasia were reported in all five cases and included status epilepticus (case 1), old age (cases 2, 4, and 10), and uncontrolled recurrent disease (case 3). The dog that was euthanized during a status epilepticus was previously diagnosed with primary epilepsy.

The reported complications in this study overlapped with complications previously described in pituitary surgery. Minor complications included mild stridor during agitation (*n* = 2), serosanguinous nasal discharge (*n* = 2), rhinitis (*n* = 2), transient hyperthermia (*n* = 3), palatal wound dehiscence (*n* = 1), mild reflux esophagitis that could be managed medically (*n* = 1), temporary (*n* = 10), and long-term electrolyte disturbances (case 7) and persistent diabetes insipidus (case 6). Major complications included aspiration pneumonia (*n* = 1), severe reflux esophagitis needing the placement of a feeding tube (*n* = 1) and central nervous signs (*n* = 1). Though tear production was decreased in all cases directly after surgery, only a single case needed long-term treatment. The sequalae of severe and ongoing keratoconjunctivitis sicca was a cause of enucleation of a single eye in that dog (case 3). The case who experienced central nervous signs had a decreased mental status at the time of admission and suffered massive bleeding at the time of surgery (case 8). The dog that was diagnosed with aspiration pneumonia developed severe upper respiratory swelling, exacerbated by the dyspnea caused by pneumonia (case 3). A temporary tracheostomy tube was placed until the pneumonia was under control, and the swelling of the upper respiratory tract subsided. Case 7 suffered from severe dehydration caused by ongoing sodium losses of unknown pathophysiology. Desmopressin did not appear to affect the sodium losses, and aldosterone levels were deemed sufficient and not the cause of the dogs' hypodipsia. In order to assess complications in the pituitary fossa, the dog had an MRI scan performed 17 days after the procedure. Imaging showed no signs of complications in the thalamic area, pituitary fossa, and hypothalamic area. This patient is currently still in remission at 695 days. The dog receives desmopressin three times a day, as without, sodium losses were uncontrollable. According to the owner, he has a good quality of life and is thriving again. Case 6 developed chronic diabetes insipidus which is well controlled by desmopressin supplementation.

Residual disease was reported in three procedures in two dogs. Two of these procedures were in the same dog (case 8). Direct postoperative plasma ACTH concentrations were indicative of residual disease in both procedures in this dog 1,207 pg/mL (prior to surgery), followed by 200 pg/mL (1 h after surgery), 153 pg/mL (2 h after surgery) and 250 pg/mL (5 h after surgery). Ongoing clinical signs after the first procedure were also indicative of persistent PDH but were considered mild. A second procedure was performed 11 months after the first, but again, postoperative plasma ACTH levels were consistent with residual pituitary adenoma. Baseline ACTH was 147 pg/mL, followed by 42 pg/mL (1 h after surgery), 66 pg/mL (3 h after surgery), and 48 pg/mL (5 h after surgery). The second case with residual disease (case 3) also showed persistent high levels of ACTH and a high postoperative UCCR. Clinical signs in this dog persisted, though initially some relief was noted.

Recurrent disease was suspected in case 6, as the UCCR 6 months post-surgery was low (1.55), but 1 year after surgery severely increased (107.75) (Table 1). Hydrocortisone acetate supplementation was tapered and eventually ceased. Clinical signs subsided, however, trilostane therapy was initiated approximately 16 months after surgery. The dog is currently still alive and clinical signs of PDH are well controlled with trilostane. Diabetes insipidus is persistent and well controlled with desmopressin administration.

Histological examination of the surgical specimens in all cases revealed a corticotrope adenoma staining immunopositive for ACTH. Five of/11 cases also showed alpha MSH immunoreactivity, and 2 of 11 cases showed GH immunoreactivity. Four cases showed an infiltrative growth pattern, of which only one case experienced recurrence. A single case (case 5) had a combined pituitary adenoma and craniopharyngeal duct cyst.

## 4. Discussion

This case study comprises a specific subset of patients within a cohort of dogs receiving a rather difficult microsurgical treatment of a complex organ with severe pathophysiological implications, combined with a distorted anatomy. The original approach through the sphenoid bone (16) is often considered difficult, as proven by the various methods described following this original procedure description, to determine the correct placement of the burr hole in mesocephalic dogs. These methods include the placement of metal markers and perioperative diagnostic imaging [Niebauer 1988 (17)], implementing a neuronavigation system (18), and more recently, the development of 3D printed patient-specific drill guides (14, 19). All these techniques may complicate and lengthen the procedure, and with only small groups and cadaveric studies available, the question arises; do the alternatives add to the safety of the procedure and outcome of the patient? The microsurgical transsphenoidal approach to the pituitary fossa as described by Meij et al. (1) is the preferred technique at our hospital. This microsurgical technique is an adaptation to the technique originally described by Markowitz et al. (16) through which the first patients at our hospital were treated (20).

The median thickness of the sphenoid bone was 6.7 mm, which is thicker than previously reported in a population of predominantly mesocephalic patients, with an average reported thickness of 4.4 mm (2). This may increase the difficulty in both directing the drill hole and pituitary fossa exploration with a ball-tipped hook. To enhance the visualization of the tumor within the fossa, the use of a videoscope on a stand can be useful (11). However, high magnification operating loupes enables free rotation of the surgeon's head thus their view in all directions which is essential for neurosurgical procedures at the microsurgical level. In pituitary microsurgery, the surgeon's view of the operating landscape should not be limited by the angular limitations of a magnifying device. Video-assisted pituitary fossa exploration may be an alternative to examining the surgical area for residual disease (7, 11). Using a handheld small-diameter endoscope with a 30–70-degree angle may aid in the exploration of the fossa with benefits including a high-resolution magnification and a better lateral view into the fossa compared to a videoscope (11) (personal data).

The approach to the pituitary fossa was often obscured by the hard palate in our cohort of brachycephalic patients. The first attempt was aborted in two cases in our cohort. Even with thorough planning, surgical landmarks may be hard to identify, and the oral cavity is crowded by soft tissues. Being aware of these anatomical differences during planning will aid in the prevention of such problems. Preoperative CT imaging-based planning is important in pituitary surgery in general, but even more crucial in brachycephalic breeds. As the anatomy of the foreshortened skull presents an extra hurdle in the approach, it is important to perform an extended CT scan to include the complete skull anatomy and hard palate. Performing a CT of the whole skull is advised, in order to perform measurements and *in silico* practice of the procedure. By visualizing the approach *in silico*, the surgeon can determine how much of the hard palate needs to be resected to reach the sphenoid bone.

The use of diagnostic imaging tools in obtaining a correct diagnosis is important as case 6 illustrates. Previous publications emphasized the use of the P/B value to determine the relative height of the pituitary gland, as the size and shape of the skull in dogs vary. The approach to determine the difference in physiological disease severity vs. pathological pituitary size in dogs has been determined to be flawed (14, 21). Another remark should be made about this case, of which there is little noted in the current literature. Case 6 was treated with trilostane, but for several months, showed progressive disease and the progression of the pituitary mass size over the course of time. In the past, in most cases that did not receive surgical intervention, pituitary surgery was not available, either due to financial or logistic constraints. With the arrival of trilostane, more patients are being treated medically, while surgery is postponed (21). Dogs with pituitary masses that are prone to growing, are being managed medically until the point they no longer respond to medical treatment. A study evaluating the effect of trilostane on the hypothalamic–pituitary axis in healthy dogs warned that the inhibition of cortisol secretion may lead to accelerating growth of pituitary corticotroph adenomas (22). Referral for surgical intervention delays leads to an increase of pituitary mass sizes offered over the course of time (4). An increase in pituitary gland size complicates surgery, recovery, and disease-free interval (4). Case 6 is a posterchild case, and we would like to emphasize calculating the P/B value and adhering to a timely referral of patients. Of all the complications listed in this study, postoperative hyperthermia and gastroesophageal reflux were not explicitly reported in previous studies (2–8). Hypothermia and hyperthermia can be caused by damage to the hypothalamus. This may arise from either a hypothalamic crisis, which is a rare complication that can occur due to pituitary pathology or surgery, and that has been described in both human medicine (23–26) and veterinary medicine (1). It can have an acute and late onset (23, 24). Neuropathic hyperthermia can also be associated with central diabetes insipidus alone (27). A hypothalamic crisis will overall have a more severe clinical presentation compared to diabetes insipidus-associated hyperthermia. Both may be transient or permanent, and the outcome will depend on concurrent clinical signs, which appear to be overall more severe in cases of neuropathic and hypothalamic hyperthermia. Supportive measures including external cooling are often contraindicated and overall, not effective, though medical treatment in the form of NSAIDS and chlorpromazine has been used with positive effects (24). However, in our group of patients, it is more likely that brachycephaly itself contributed to the reported hyperthermia as this is a relatively common complication in hospitalized brachycephalic dogs. All patients that experienced either a single or multiple episodes of hyperthermia, did respond well to supportive measures including external cooling, sedation if associated with excitation, and intravenous fluid therapy.

Transient nasal discharge or rhinitis was seen in four cases. Nasal discharge and rhinitis have been previously reported and are generally considered to be a sequalae of the healing process of the nasopharynx. This may be predisposed by damage to the palatine artery which may be lacerated by the shortening procedure of the hard palate. In one case (number 9), rhinitis was associated with a full-thickness dehiscence of the palatal incision, which also has been previously reported (5, 28–30). None of the cases appeared to have long-term complications after hard palate resection, based upon the evaluation of clinical signs at the time of follow-up. The healing process of the nasopharyngeal soft tissues overlying the defect in the hard palate has not been assessed in this study. Second look rhinoscopy or CT scan could be useful in cases that have refractory upper respiratory complaints including rhinitis. Damage within the nasopharynx could lead to clinical nasopharyngeal stenosis or other upper respiratory dysfunctions; however, this was not suspected in our cases. Even though the nasopharyngeal mucoperiosteum was not sutured and not recognized anymore, the mucosal defect likely scarred over or epithelialized. In comparison, the mucoperiosteum overlying the sphenoid burr hole is often only partially sutured. The remnants of the mucoperiosteum rarely completely cover the bone wax filling in the osseous defect. Occasionally, in dogs in which a second hypophysectomy procedure is performed, e.g., in the instance of case 8, the mucoperiosteum was healed over the defect. We expect the same to happen with a mucoperiosteal defect on the inside of the hard palate defect. Rhinoscopy may provide a better evaluation in non-clinical cases, as the mucoperiosteum covering the hard palate within a normal nasopharynx is very thin and hard to assess on a CT scan.

One dog developed pneumonia during hospitalization, which is a known complication in brachycephalic dogs undergoing anesthesia (31) and dogs undergoing transsphenoidal hypophysectomy (1, 4). A retrospective study assessing 243 brachycephalic dogs in a case–control study, found that 4% of brachycephalic dogs developed pneumonia as a sequalae of an anesthetic procedure, compared to 0% in the non-brachycephalic control group (31). They also found a significant increase in stertorous breathing (1.8% for brachycephalic dogs compared to 0.4% for non-brachycephalic dogs). Brachycephalic dogs were also at risk for regurgitation with a reported prevalence of 3.1% of the population brachycephalic dogs (31), a well-reported complication that we also have seen in our patient population in this study (31–33).

At the time of surgery, no additional procedures to address concurrent upper airway pathologies were performed besides inspection. As the surgical procedure already penetrates the soft palate, more trauma to the soft tissues by the resection of a portion of the soft palate could lead to even more postoperative swelling. Most dogs either had only mild clinical signs of brachycephalic obstructive airway syndrome (BOAS) or had prophylactic upper respiratory procedures performed at a much earlier stage in life. One dog (case 5) had a staphylectomy and alar fold plasty performed during the same anesthesia of the transsphenoidal hypophysectomy. The necessity of prophylactic surgery in dogs with mild clinical signs is a controversial topic. Where recent studies showed that elective upper respiratory surgery in younger dogs can be associated with more complications (33, 34); previous corrective surgery may decrease the risk of complications in subsequent anesthetic procedures (35), suggesting that early intervention may be advised in cases in which multiple anesthetic procedures are required. Another a large retrospective study with 248 dogs, found that with increasing age, dogs suffer from more complications when undergoing upper respiratory surgery (36). In our opinion, the choice to perform such procedures should be based on a patient-by-patient assessment. This may include the severity of clinical signs associated with BOAS, the effect of prolonging the overall anesthesia length when combining these procedures with the iatrogenic surgery, comorbidities, or proximity of the primary surgical procedure (32). If surgical intervention is necessary, the upper respiratory surgery should preferably not be combined with the pituitary procedure. Prolonged anesthesia in dogs with very large pituitary glands may not be preferable, and severe and uncontrolled PDH may give rise to problematic and prolonged wound healing. It may be more prudent to combine preoperative imaging with upper respiratory surgery or perhaps postpone any other surgical interventions until the dog has fully recovered from the transsphenoidal hypophysectomy. When a potential patient with BOAS is presented for transsphenoidal hypophysectomy, assessment of upper airways and intervention is advised. The CT scan performed for surgical planning can aid in identifying excessive aberrant turbinates, while an oral inspection at the time of the CT or MRI scan should be performed. Assessment of elongated or thickened soft palate, tonsillar eversion, and laryngeal collapse should be staple practice in any brachycephalic dog undergoing anesthesia and should be addressed accordingly. The treatment team should also be prepared for intervention after surgery. Interventions may include adrenaline nebulization in mild to moderate cases of upper respiratory swelling (37), and tracheostomy tube placement (38) and/or postoperative mechanical ventilation in severe cases of lower and upper airway respiratory complications (39). These complications and interventions should be discussed with the client as part of the PDH treatment plan.

A pertinent question to still be answered is whether all dogs with severe brachycephaly are eligible for transsphenoidal hypophysectomy procedure. The surgical technique can be altered to the patients' specific anatomy as specified above. In dogs with severe BOAS, repeated anesthetic procedures, as needed for radiation therapy, may be equally or more prone to complications (32–36). Radiation therapy also does not immediately aid in the decompression of the brain in larger masses, while surgery does have this additional benefit (40, 41). Medical management using trilostane may accelerate pituitary tumor growth over time, adding to the risk of compressive effects of the mass on the brain (22). The choice of treatment depends on many factors. As with all dogs, the benefits of the treatment should be weighed against the risks. However, comorbidities are common in severe brachycephalic dogs. In addition to BOAS, spinal diseases such as disk herniation and subarachnoid diverticulae, and ophthalmologic diseases including chronic corneal ulceration, keratoconjunctivitis sicca, and primary glaucoma, may be present in a single patient. These comorbidities should be recognized, evaluated, and managed prior to surgery in order to minimize their impact on the patients' quality of life and recovery from the hypophysectomy procedure.

One case (case 7) developed severe and persistent hyponatremia which we were not able to explain. The presence of postoperative transient hypernatremia was recorded and was treated as described previously (10). Another case (case 6) had persistent diabetes insipidus which is managed by desmopressin suppletion.

Postoperative mortality and remission in dogs in this study seem similar to data reported for the total cohort (4), but the group is too small to perform statistical analysis. Within our small cohort, we reported one recurrent disease (case 6) and three occasions of persistent disease. In case 6, recurrent disease became evident after 1 year post-surgery and was supported by both the recurrence of clinical signs of polyuria/polydipsia and an increased UCCR. In case 3, the postoperative UCCR at 4 and 8 weeks were relatively high, though still below 10. Postoperative ACTH remained high, suggestive of residual disease (Table 1). Persistent high levels of ACTH were also found after two procedures in the same dog (case 8). Direct postoperative serum ACTH concentrations were indicative of residual disease in both procedures in this dog. During the first procedure, a cystic lesion and mucoid-like adenomatous tissue were removed from the pituitary fossa, and although no macroscopic tissue was left behind, large, very friable tissue can cause local seeding during extraction. At the time of the second procedure in this dog, profuse hemorrhage obscured the surgical field, making it hard to assess the completeness of the resection. This may have contributed to the incomplete excision. There was, however, also an invasive growth pattern in histopathology. By the nature of the pituitary location, surgical excision of a pituitary mass will always be a marginal excision, making the procedure prone to failure in cases of infiltrative pituitary disease. The initial mass in this dog had a P/B value of 0.75. These very large pituitary tumors do have a larger chance of recurrent disease (4). The median size of the pituitary glands removed in this study is large compared to the general population, with a median P/B value of 0.5. Van Rijn et al. (4) already reported a significant increase in pituitary size over time in their 2016 study including 257 dogs that received surgery over the period of 1993–2013. The spread of these particular cases does not show such a distribution over time. Residual disease has been reported at 8.3% (1997), 6.6% (2007), and 5.7% (2016) in previous reports (2, 4, 7). This gradual decrease in residual disease over time and increase in general pituitary size appears to show a learning curve/increase in dexterity combined with an increase in difficulty over time.

An infiltrative growth pattern was found on histopathology in four cases (Table 2), of which only one case (case 8) developed local tumor recurrence. In that specific case, not all tumor tissue was removed, as postoperative ACTH levels remained high, although the dog remains in clinical remission. The other three cases remained free of disease over 36, 7, and 6 months. A small case series compared imaging results with histopathology and found that older dogs with larger tumors were more prone to develop invasive adenomas. Unfortunately, most samples were taken postmortem (42). The clinical relevance of the invasive nature of the masses in dogs is to be determined. These types of adenomas may be completely excised, as has been described in a human case series on the dural invasion of pituitary adenomas (43). Though our sample size is small, we do conclude that the infiltrative nature of some of these tumors may not be followed by recurrent disease.

Case 5 in this small retrospective was diagnosed with a combined pituitary adenoma and a craniopharyngeal duct cyst. Cysts within the pituitary gland are a rare entity and there is only one reported Radke's cleft cyst in the veterinary literature. These cysts may cause a non-functional enlargement of the pituitary gland and may only present with clinical signs after severe enlargement causes central nervous signs (44) or as an obstruction of the nasopharynx (personal data).

## 5. Conclusion

Transsphenoidal hypophysectomy in brachycephalic dogs can be safely performed with several precautionary measures. It is recommended to prepare the brachycephalic dog for hypophysectomy using a protocol that includes preoperative assessment and correction of BOAS issues that are expected to have a difficult recovery and to take into account immediate post-hypophysectomy procedures such as tracheostomy or laryngeal stenting when the need arises. Preoperative *in silico* planning based on CT reconstructions, intraoperative shortening of the hard palate, and identifying all surgical landmarks in relation to the location of the pituitary gland enables the placement of an accurate burr hole to approach the pituitary fossa safely. In addition, specialized anesthesia ology and intensive care addressing general brachycephalic pathophysiology in the perioperative period are imperative for a good outcome. Resection of part of the hard palate did enable proper access to the sphenoid bone and planned burr hole and did not increase morbidity.

## Data availability statement

The original contributions presented in the study are included in the article/supplementary material, further inquiries can be directed to the corresponding author.

## Author contributions

LV and BM contributed to the conception and design of the manuscript and organized the database for this specific study purpose. BM, SG, SV, and LV performed data collection and follow-up. LV performed the statistical analysis and wrote the first draft of the manuscript. LV, SV, and BM wrote sections of the manuscript. All authors contributed to the manuscript revision, read, and approved the submitted version.
